# Expression and significance of Fractalkine/CX3CL1 in MPO-AAV-associated glomerulonephritis rats

**DOI:** 10.1186/s12882-024-03565-3

**Published:** 2024-06-27

**Authors:** Junxue Ma, Junjie Wang, Hongli Kang, Ruiying Ma, Zhengxi Zhu

**Affiliations:** 1 Department of Nephrology, The people’s hospital of Baise, Baise, China; 2 Department of Nephrology, Cangxi People’s Hospital, Cangxi, Sichaun China; 3 Dapartment of Emergenc, The Third Hospital of Shijiazhuang, Shijiazhuang, China; 4 Department of Nephrology, The Affiliated Hospital of Youjiang Medical University for Nationalities, Baise, China

**Keywords:** Fractalkine, MPO-AAV, Glomerulonephritis, Expression levels

## Abstract

**Objective:**

To investigate the expression and significance of Fractalkine (CX3CL1, FKN) in serum and renal tissue of myeloperoxidase and anti-neutrophil cytoplasmic antibody associated vasculitis (MPO-AAV) rats.

**Methods:**

Thirty Wistar-Kyoto (WKY) rats were randomly divided into: Control group, MPO-AAV group (400 µg/kg MPO mixed with Freund’s complete adjuvant i.p), MPO-AAV + Anti-FKN group (400 µg/kg MPO mixed with Freund’s complete adjuvant i.p), anti-FKN group (1 µg/ rat /day, i.p) after 6 weeks. MPO-AAV associated glomerulonephritis model was established by intraperitoneal injection of MPO + Freund’s complete adjuvant with 10 mice in each group. The concentration of MPO-ANCA and FKN in serum was detected by Enzyme-linked immunosorbent assay (ELISA). Hematoxylin-eosin (HE) staining was used to detect pathological changes of kidney tissue. Western blot and immunofluorescence staining were used to detect the expression and localization of FKN protein in kidney tissue. Renal function test indicators: 24-hour urinary protein (UAER), blood urea nitrogen (BUN), serum creatinine (Scr). The expression levels of p65NF-κB and IL-6 was detected by Immunohistochemical assays.

**Results:**

Compared with the control group, the serum MPO-ANCA antibody expression level in the MPO-AAV group was significantly increased (*P < 0.01*), and the contents of UAER, BUN and Scr were significantly up-regulated at 24 h (*P < 0.01*). Compared with the control group, the glomeruli in the MPO-AAV group had different degrees of damage, infiltration of inflammatory cell, and membrane cell hyperplasia and renal tubule edema. Compared with the control group, rats in the MPO-AAV group had significantly higher levels of FKN in serum and renal tissues (*P < 0.01*), and high expression of p65NF-κB and IL-6 in renal tissues (*P < 0.01*) (*P < 0.05*), whereas anti-FKN reversed the expression of the above factors. In MPO-AAV renal tissue, FKN was mainly expressed in the cytoplasm of renal tubular epithelial cells and glomerular podocytes. In addition, the contents of 24 h UAER, BUN and Scr of renal function in MPO-AAV rats were significantly decreased (*P < 0.01*) and the damage of renal tissue was significantly ameliorated after the administration of antagonistic FKN.

**Conclusion:**

FKN may play a key role in the pathogenesis of MPO-AAV associated glomerulonephritis.

**Supplementary Information:**

The online version contains supplementary material available at 10.1186/s12882-024-03565-3.

## Introduction

Antibodies Anti-Neutrophil Cytoplasmic Antibodies (ANCA) associated Vasculitis (ANCA-associate vasculitis, AAV) is a systemic autoimmune disease mainly characterized by inflammation of multi-system and multi-organ small vessels and fibrinoid necrosis [1, 2]. Anti-neutrophil cytoplasmic autoantibodies against circulating myeloperoxidase (MPO) are associated with systemic small vasculitis, typically characterized by necrotic crescent nephritis (NCGN) [3–5]. LITTLE et al. mixed purified MPO with the Freund’s complete adjuvant and gave the immunoWistar-Kyoto (WKY) rat abdominal injection to establish the ANCA-related experimental autoimmune vasculitis (the autoimmune vasculitis, EAV) rat model [6] to produce high-titer anti-MPO antibodies that cross-react with rat MPO. After 6 weeks, the model Wistar-Kyoto rats showed pathological changes of glomerulonephritis and pulmonary capillary vasculitis similar to human AAV, so it was called ANCA-associated AAV rat model. At the same time, this model also confirmed that ANCA could lead to vascular endothelial injury by enhancing the adhesion and transfer of neutrophils and the cytotoxic effect on endothelial cells, and lead to celluline-like necrosis of small vessels, which was consistent with the results of neutrophils incubated with anti-MPO antibodies in vitro eventually [7]. Therefore, this study will refer to previous methods to construct MPO-AAV rat model for experimental study.

Neutrophils are the main effector cells in disease induction [8] because the absence of neutrophils prevents the progression of the disease completely [9]. The recruitment process of inflammatory cells to the inflammatory sites of tissues is regulated by chemokines to a large extent [10–12]. Chemokines are secreted by activated or damaged cells that are recognized by specific G-protein-coupled receptors expressed on white blood cells. According to the position of the first two cysteines in the conserved amino acid sequence, chemokines can be divided into four families (C, CC, CXC, and CX3C). Most chemokines belong to the CXC chemokines family, which recruit neutrophils, T cells, and B cells, or the CC chemokines family, which recruit multiple subsets of white blood cells, including monocytes and T cells, but not neutrophils. CXC chemokines containing the ELR^+^ (glutamine-leucine-arginine) motif are particularly effective against neutrophils. FKN/CX3XL1 is a unique member of the CX3C-chemokine-like subfamily. Compared with other chemokines, FKN/CX3XL1 exists in both membrane-bound and soluble forms, which mediate different biological actions. Membrane binding FKN is mainly expressed in activated fibroblasts, endothelial cells and osteoblasts, mediating adhesion. However, soluble FKN has strong chemotaxis, especially against T cells and NK cells. In addition, FKN/CX3CL1 also has biological functions such as regulating cell growth and participating in inflammatory and immune responses [13–15]. More and more data confirm that CX3CL1/CX3CRI is involved in various immune inflammatory diseases, such as glomerulonephritis, rheumatoid arthritis, and systemic lupus erythematosus [16–19]. Many studies have also confirmed that FKN/CX3CL1 plays a key role in the pathogenesis of ANCA-associated vasculitis [20, 21]. However, there are few reports on the expression, localization and function of FKN in MPO-AAV. A previous study has confirmed that the expression level of FKN in patients with medium and small vasculitis is higher than that of healthy people, and the expression level of FKN in patients with ANCA-associated vasculitis is higher than that in patients with other types of vasculitis, and the expression level is highest in patients with multiple vasculitis under the microscope [21]. It has also been found that the expression level of FKN is positively correlated with Birmingham vasculitis activity fraction, erythrocyte sedimentation rate and C-reactive protein level, and decreases with the relief of clinical symptoms after drug treatment [22]. In this study, we investigate the expression of Fractalkine(CX3CL1,FKN) in serum and renal tissue of myeloperoxidase and anti-neutrophil cytoplasmic antibody associated vasculitis (MPO-AAV) rats and to further explore theand significanceof FKN in this disease.

## Materials and methods

### Experimental animals

**Fig. 1:**
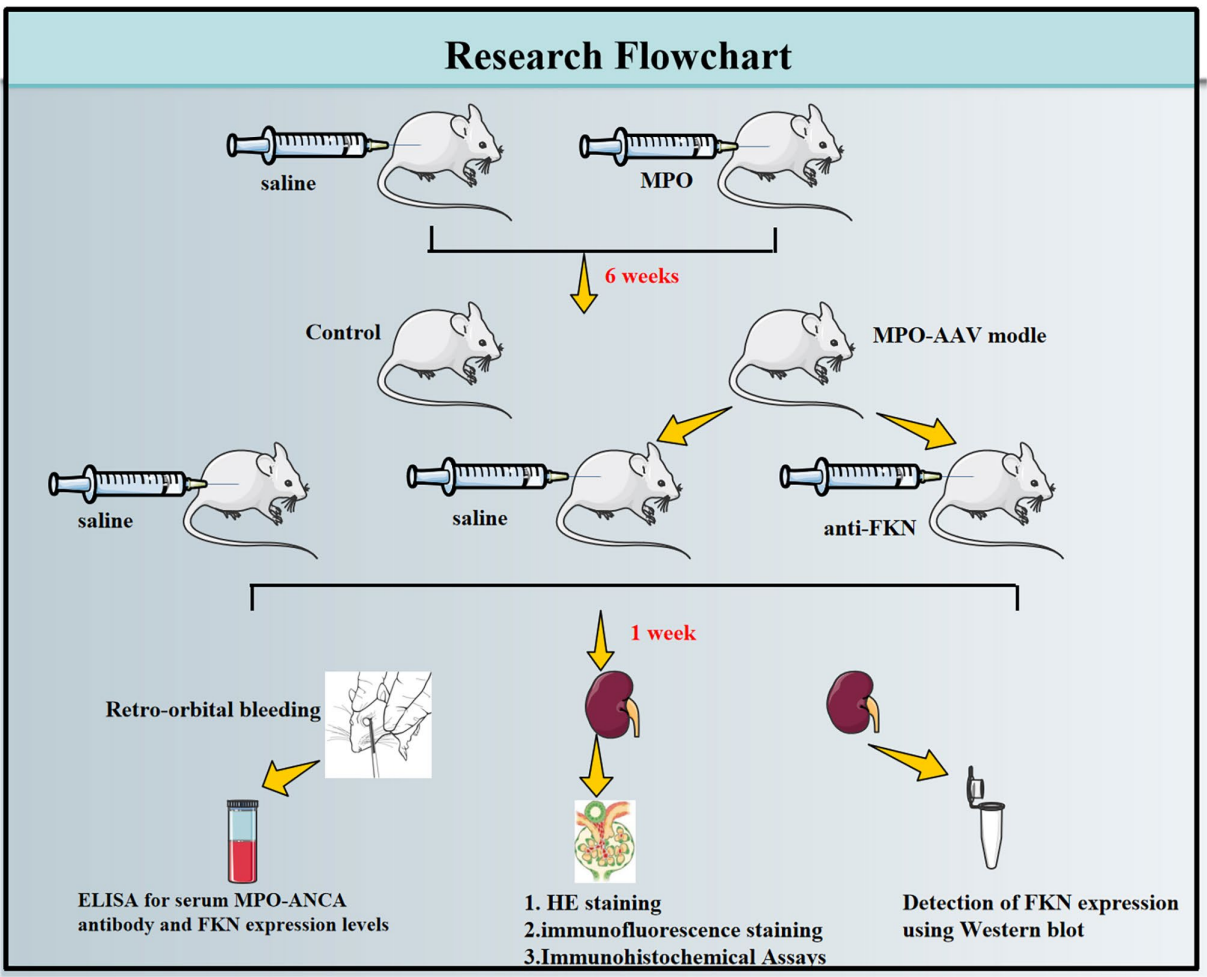
Schematic illustration of the experimental procedures. Myeloperoxidase(MPO), myeloperoxidase and anti-neutrophil cytoplasmic antibody associated vasculitis (MPO-AAV), anti-fractalkine (anti-FKN)

Figure 1 illustrates the experimental and workflow of FKN detection. WKY rats were purchased from Changsha Tianqin Biotechnology Co., LTD., Hunan. Myeloperoxidase (MPO, Sigma-Aldrich, M6908-5UN), Freund’s complete adjuvant (Sigma-F5881), MPO-ANCA-IgG ELISA Kit (CB-E08675R; Cusabio Ltd., Wuhan, China), Anti-rat Fractalkine neutralizing antibody (AF537; R&D Systems), Fractalkine ELISA kit (Bethyl Laboratory), Antibodies against Fractalkine ( DF12376, Affinity Biosciences, Changzhou, China). Anti-glyceraldehyde3-phosphatedehydrogenase (GAPDH; AF7021, Affinity Biosciences, Changzhou, China). Creatinine(Cr)Colorimetric Assay Kit (Sarcosine Oxidase Method, E-BC-K188-M, Elabscience Biotechnology Co.,Ltd, Wuhan, China; Urea (BUN) Colorimetric Assay Kit (Ur, ease Method, E-BC-K183-M, Elabscience Biotechnology Co.,Ltd, Wuhan, China); Urine Protein Colorimetric Assay Kit(E-BC-K252-M, Elabscience Biotechnology Co.,Ltd, Wuhan, China).

### Establishment of animal models and collection of tissue specimens

Thirty Wistar-Kyoto (WKY) rats were randomly divided into three groups (*n* = 10/group) the control group; the MPO-AAV group in which rats were given a one-time intraperitoneal injection of 400 µg/kg MPO (MPO dissolved in sterile water for injection at a concentration of 500 µg/ml, then mixed with an equal amount of Freund’s complete adjuvant [23–27]; and the MPO-AAV + anti-FKN group, in which rats were given a single intraperitoneal injection of 400 µg/kg MPO for 6 weeks, then anti-FKN (1 µg/ rat /day, i.p) [28, 29]. One week later, orbital blood was collected on EDTA-K2 anticoagulant tube, and the rats were killed by disjoint method. The abdominal cavity was fully exposed, both kidneys were removed, half kidney tissue was cut open, half kidney tissue was fixed with 10% formalin, paraffin embedding was used for immunofluorescence staining and HE staining, and the other half kidney tissue samples were extracted and stored at -80℃ for total protein extraction.

### Detection of renal function indicators

According to related kit instructions, urinary albumin (UAER), serum creatinine (Scr) and blood urea nitrogen (BUN) contents in urine were detected by automatic biochemical analyzer.

### Enzyme-linked immunosorbent assay

The levels of MPO-ANCA, and FKN in rat serum were detected using the respective enzyme-linked immunosorbent assay kits. The absorbance was measured at 450 nm using a microplate reader.

### Western blot assay

The total protein of rat kidney tissue was extracted based on previous research methods [30], the protein concentration was detected by BCA method, the protein sample was added for SDS-PAGE electrophoresis, the membrane was transferred at a constant voltage of 100 V for 110 min, the protein was transferred to the nitrocellulose membrane, and the sealing liquid was used for 60 min. Anti-FKN (DF12376), 1:1100) (Affinity Biosciences, Changzhou,, China) was added and incubated at 4℃ overnight. After Tris-HCI Tween(TBST) washing for 3 times, corresponding horseradish peroxidase labeled secondary antibody was added (1: 2000), incubated in a shaker at room temperature for 50 min, enhanced chemiluminescence (ECL) development was performed. ImageJ software was used for semi-quantitative analysis, and the relative content was represented by the ratio of gray values of target protein bands to GAPDH protein bands. The experiment was repeated three times.

### Immunofluorescence staining

Paraffin sections of kidney tissue were fully dewaxed by xylene, 100%-75% gradient alcohol to water, then boiled in EDTA buffer solution (pH8.0) for 20 min, washed 3 times in PBS after natural cooling, and incubated at room temperature for 1 h in the closed solution (containing 2% BSA and 10% goat serum). Then FKN monoclonal antibody was incubated overnight, followed by PBS for 3 times on the second day, and the corresponding fluorescent secondary antibody was incubated for 1 h. After PBS for 3 times, DAPI was incubated for 10 min and nucleated. After PBS cleaning, the tablets were sealed with anti-fluorescent attenuation tablets. The FV3000 laser scanning confocal microscope was used to obtain images of 5 fields per rat.

### Hematoxylin-eosin staining

The renal tissue sections were stained by Thermo scientific GeminiAS, sealed by Clearvue automatic slice sealing machine, scanned by 3DHistech Pannoramic Midi scanner, and collected by caseviewer software.

### Immunohistochemical assays

Single-labelled immunohistochemical assays were performed using p65NF-κB and IL-6 primary antibodies. Sample slides were incubated with the corresponding primary antibodies overnight at 4 °C, then labelled with secondary antibodies (goat anti-rabbit, PV-6000, ready-to-use, ZSBG-BIO, China) for 20 min at 37 °C. PBS was rinsed, and the labelled tissues were labelled with freshly prepared DAB chromogenic reagent. The reaction time was observed with a microscope until the positive expression showed brownish yellow colour. The nuclei were stained with hematoxylin staining solution (BA-4041, BASO, China). The slides were then treated with differentiation solution (C0163M, Beyotime, China) for a few seconds and washed with counterblue in running tap water. As identified by DAB reagent (ZLI-9018, ZSBG-BIO, China), the cells of positive cells were brownish-yellow in colour and the nuclei were blue.

### Statistical analysis

SPSS 23.0 statistical software was used for data analysis. All measurement data were expressed as X ± S, and data were analyzed by means comparison of multiple samples and pair comparison. α = 0.01 was used as the test level, and the homogeneity test of variances was conducted. *P* < 0.01 was considered statistically significant.

## Results

### The rat model of MPO-AAV associated glomerulonephritis was established successfully

**Fig. 2:**
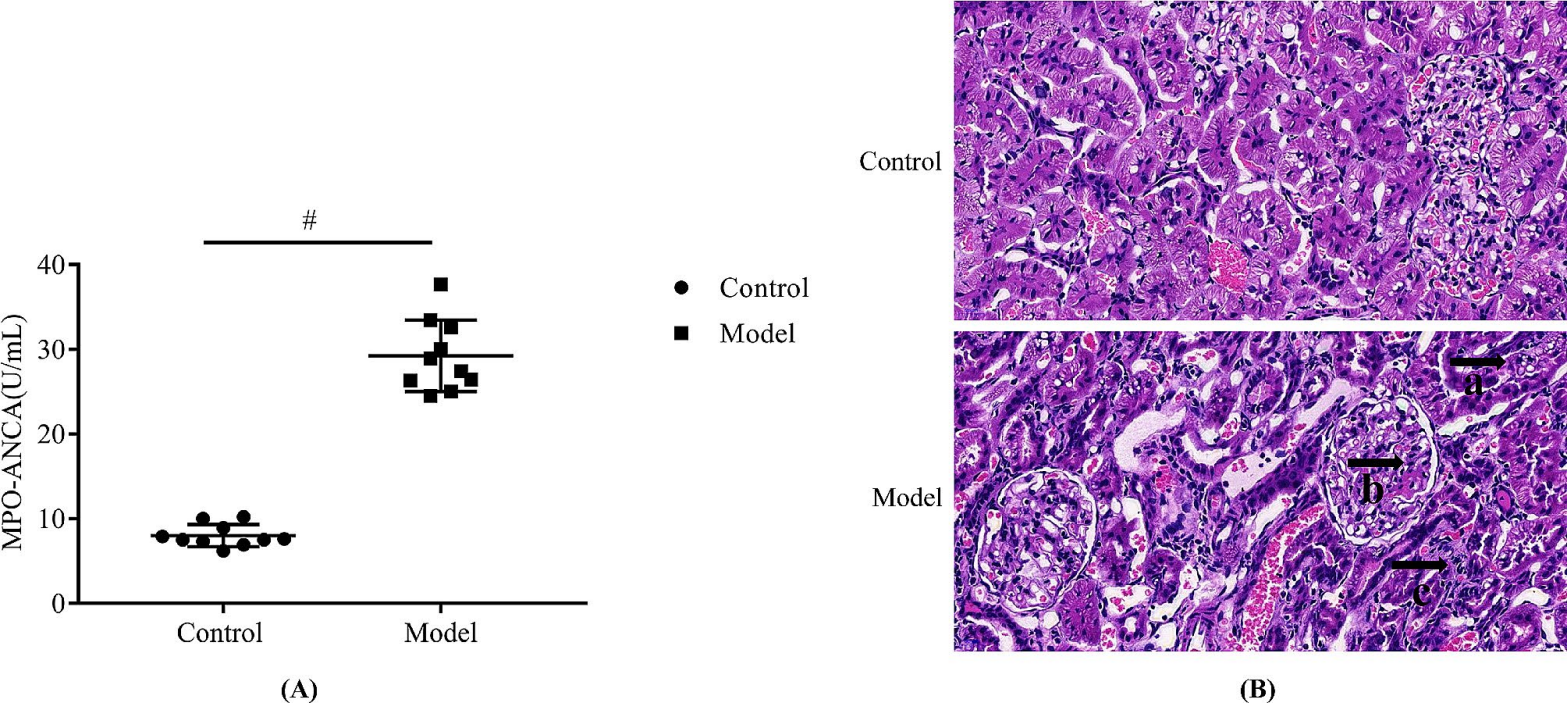
Validation of a rat model of MPO-AAV-associated glomerulonephritis. (**A**) serum MPO-ANCA antibody levels were measured by ELISA, and (**B**) renal tissue changes were observed by HE staining (x20). **a**. vacuolar degeneration of renal tubules, **b**. thylakoid proliferation, **c**. iInflammatory cell infiltration

Figure 2 shows that serum MPO-ANCA content in the MPO-AAV group was significantly higher than that in the control group (*P < 0.01*). (Moreover, compared with the control group, the glomeruli of the model rats had varying degrees of injury, inflammatory cell infiltration, mesangial cell proliferation, and tubular edema. These results indicate that a rat model of MPO-AAV associated glomerulonephritis was successfully established.

### Expression of serum FKN protein

**Fig. 3:**
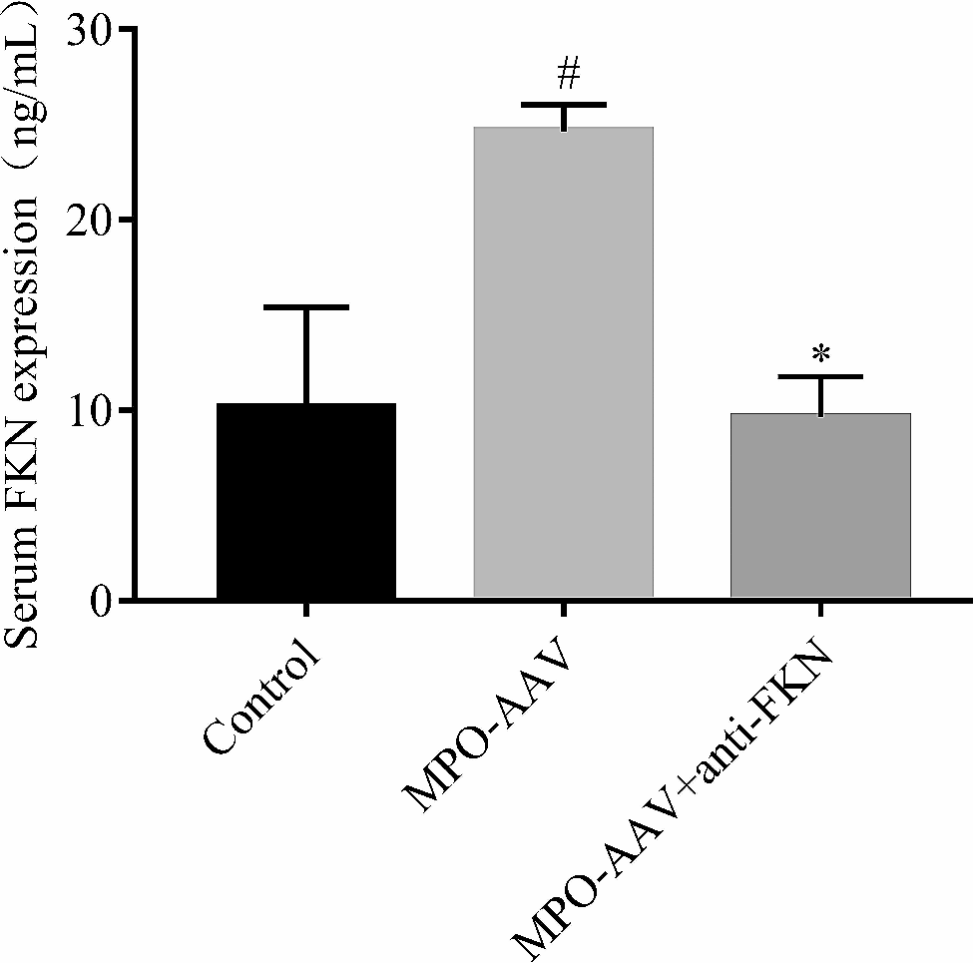
The concentration of FKN in serum of rats was detected by ELISA. ^*#*^*P < 0.01*, compared with MPO-AAV group; ^*^P > 0.05, compared with the control group

The protein expression of FKN was analyzed in rat serum by ELISA. Compared with the control group (10.141 ± 5.28321), the level of FKN protein in the serum of the MPO-AAV group was significantly increased (24.6370 ± 1.3904) ng/ml (*P < 0.01*) (Fig. 3). Compared with the MPO-AAV group, the level of serum FKN protein in the MPO-AAV + Anti-FKN group was significantly decreased (9.6460 ± 2.1208) (*P < 0.01*), and the difference was statistically significant. However, compared with the control group, the serum FKN protein level of the Anti-FKN group had no significant change (*P > 0.05*), and the difference was not statistically significant (Fig. 3).

### Expression of FKN protein in kidney tissue

**Fig. 4:**
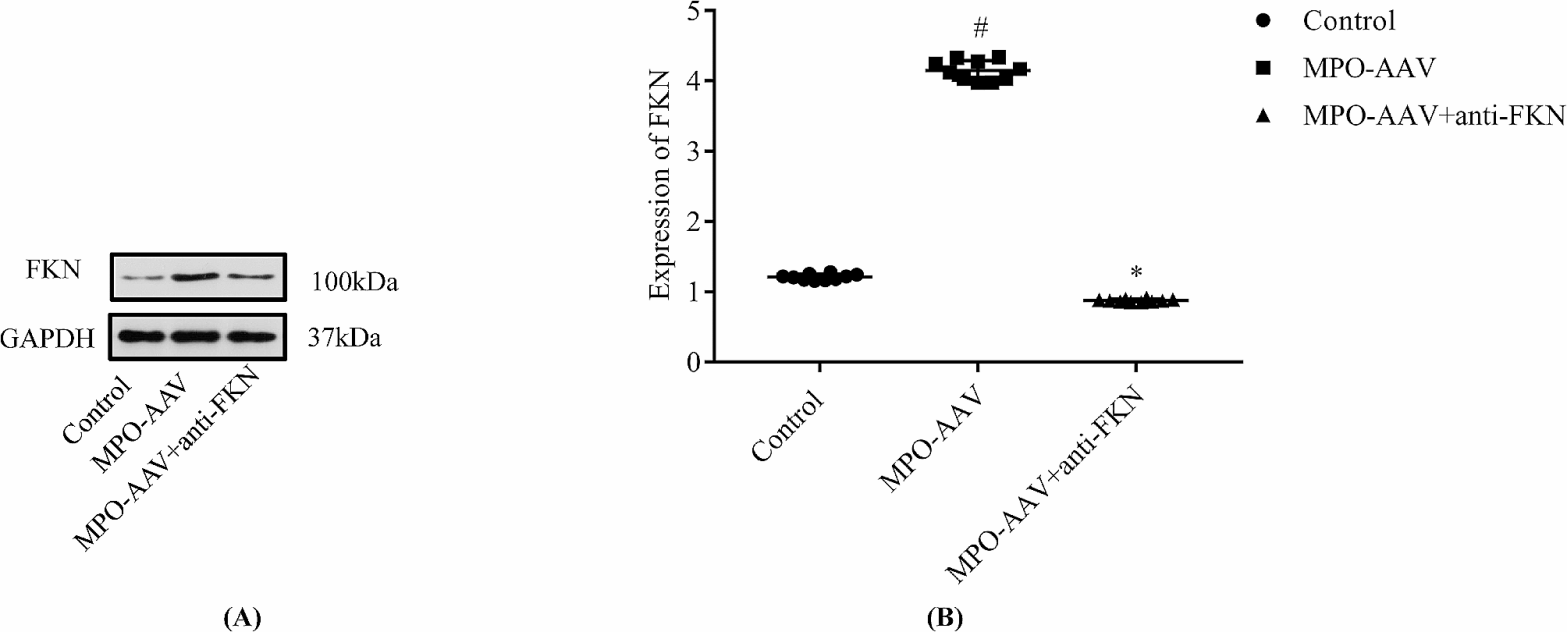
FKN expression in rat kidney tissue detected by Western blot (*n* = 10) (**A**). (**B**)^*#*^*P < 0.01*, compared with MPO-AAV group; ^*^P > 0.05 compared with normal control group

As observed in Fig. 4, the renal FKN protein expression level in the MPO-AAV group was increased significantly compared with the control group (*P < 0.01*). Additionally, compared with the MPO-AAV group, the expression level of FKN protein in the MPO-AAV + Anti-FKN group was decreased significantly (*P < 0.01*). However, compared with the control group, there was no significant change in FKN protein expression in the MPO-AAV + Anti-FKN group (*P > 0.05*).

### Localization and expression of FKN in renal tissue

**Fig. 5:**
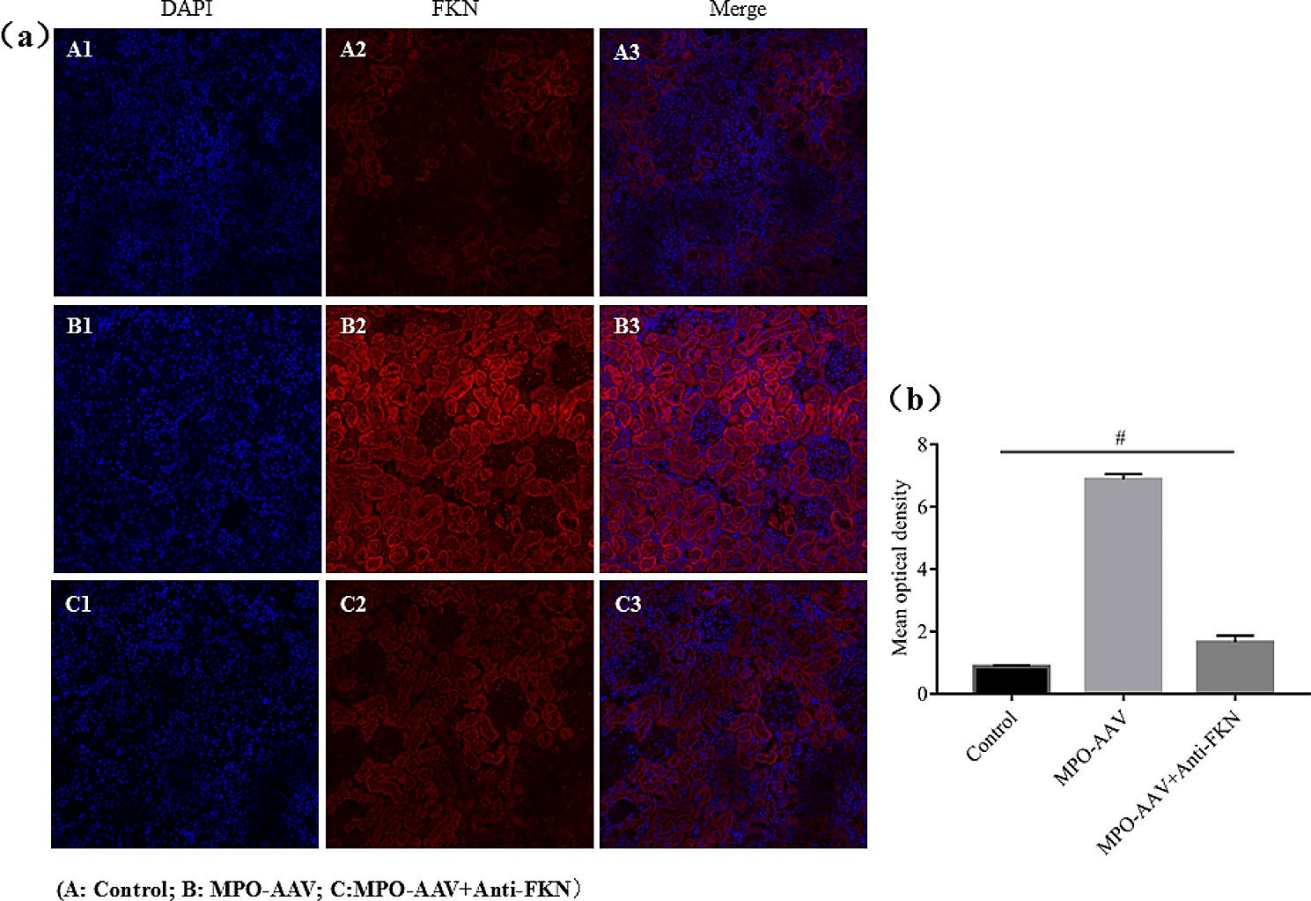
Immunofluorescence staining to detect the expression and localization of FKN in rat kidney tissue. (**a**) FKN was mainly expressed in renal tubular epithelial cells and glomerular cytoplasm (x20). (**b**) ^*#*^*P < 0.01*, compared with MPO-AAV group

The results of immunofluorescence chemical staining (Fig. 5) showed significantly higher FKN expression in the renal tissues of MPO-AAV compared with the control group. This expression (red fluorescence) was mainly in the cytoplasm of renal tubular epithelial cells and glomerular podocytes. Semi-quantitative analysis showed that compared with the control group (0.8762 ± 0.3272), the mean optical density of FKN in renal tissue of MPO-AAV group was significantly increased (6.8822 ± 0.1681) (*P < 0.01*). Compared with the MPO-AAV group, the average optical density of FKN in renal tissue of the MPO-AAV + Anti-FKN group was significantly decreased (1.6591 ± 0.2080) (*P < 0.01*). However, compared with the control group, there was no significant difference in the average optical density value of the MPO-AAV + Anti-FKN group (*P > 0.05*) (Fig. 4).

### Antagonistic effect of FKN can restore renal function and renal tissue damage in MPO-AAV rats

**Fig. 6:**
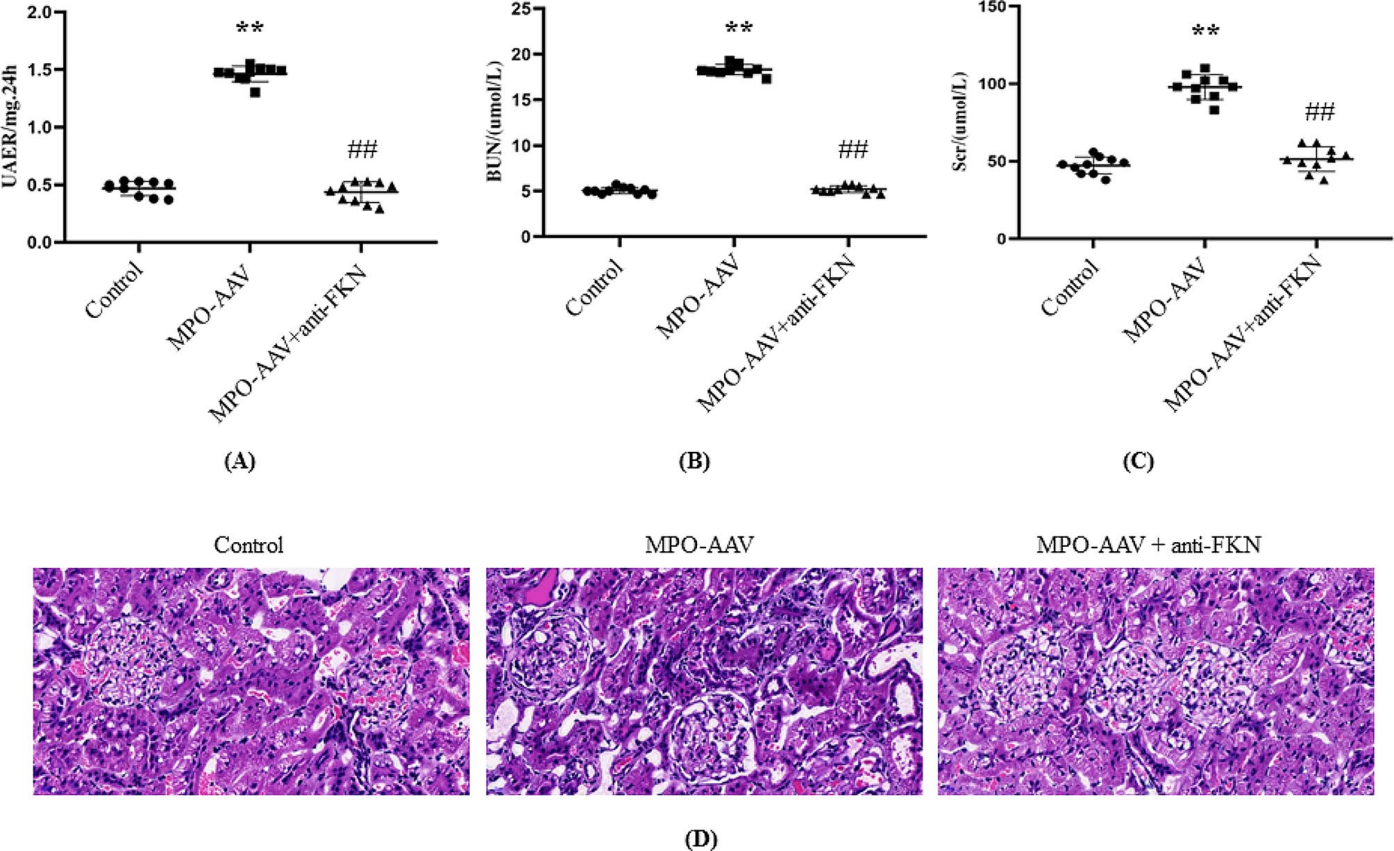
Effect of antagonistic FKN on renal function indexes and renal tissue in MPO-AAV rats. Renal function indexes (**A**: UAER, **B**: BUN, **C**: Scr, *n* = 10) were detected.^**^P < 0.01, compared with normal control group; ^*##*^*P < 0.01*, compared with MPO-AAV group. (**D**) pathological changes of kidney tissue in normal group, MPO-AAV group and MPO-AAV + anti-FKN group (x400). a.vacuolar degeneration of renal tubules, b.proliferation of thylakoid stroma and thylakoid cells, c. inflammatory cell infiltration

By exploring the effect of antagonistic FKN on renal function indexes of MPO-AAV rats (Fig. 6), it was found that compared with the control group, the levels of UAER, BUN and Scr in MPO-AAV group were elevated significantly (*P < 0.01*). After antagonizing FKN, the levels of UAER, BUN and Scr in rats were decreased significantly (*P < 0.01*). Besides, compared with the control group, the renal tissue of MPO-AAV rats was seriously injured, the glomeruli are ischemically constricted to varying degrees, with increased glomerular capillary stenosis and occlusion of the thylakoid matrix, thylakoid cell hyperplasia, basement membrane thickening, and interstitial inflammatory cell infiltration in the renal interstitium. Compared with the MPO-AAV group, the degree of renal tissue damage in the MPO-AAV + Anti-FKN group was significantly recovered.

**Effect of antagonism of FKN on the expression of inflammatory pathway factors p65 NF-κB and IL-6 in MPO-AAV rat renal tissues.**

**Fig. 7:**
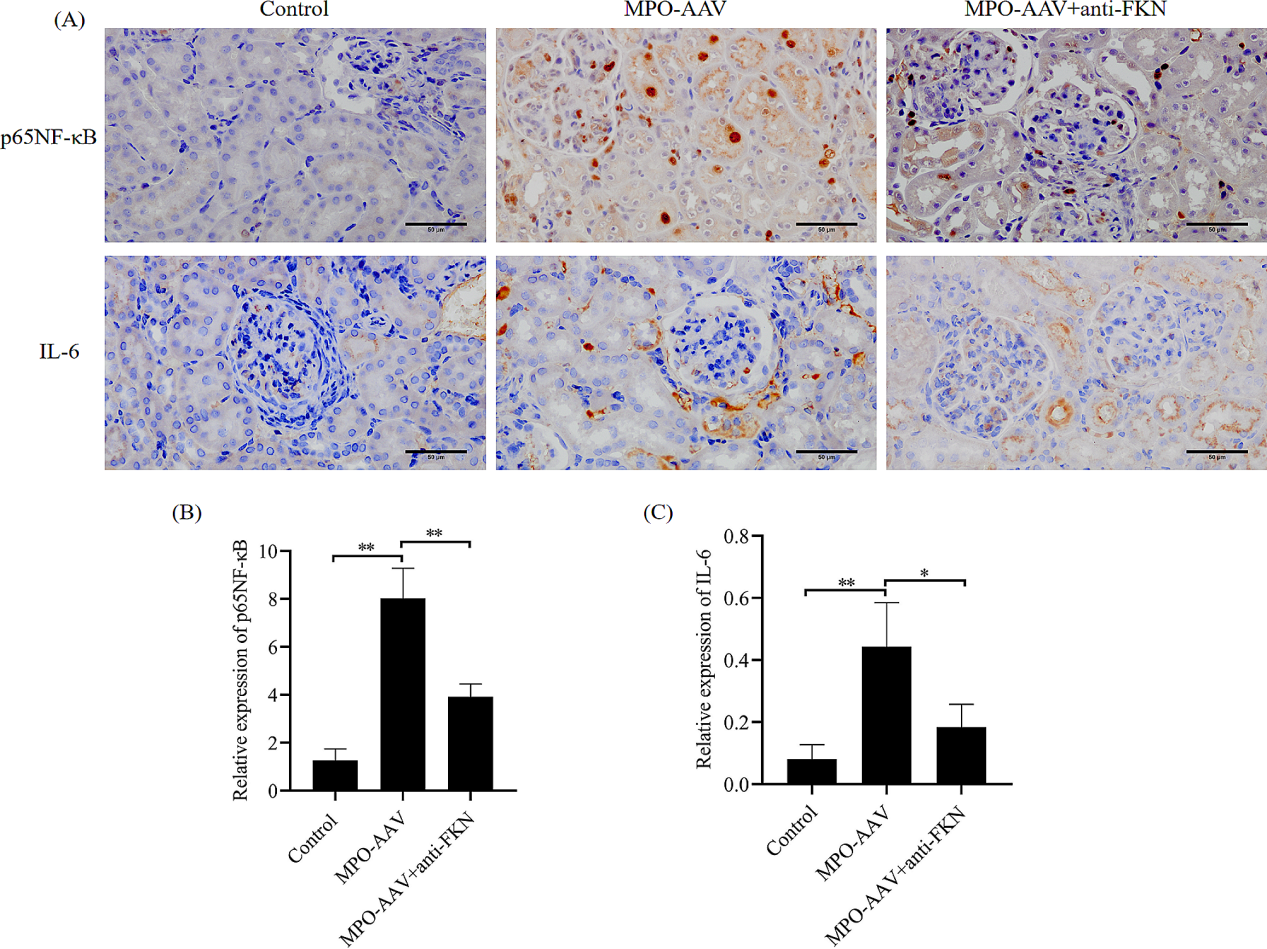
(**A**) Immunohistochemistry was detect the expressions of NF-κB P65 and IL-6 in rat kidney tissue (x400). (**B-C**)^**^P < 0.01, compared with control group,^*^P < 0.01, compared with MPO-AAV group

To further evaluate the effect of anti-FKN on the inflammatory pathway NF-kB in the injured kidney, immunohistochemistry was used to detect the expression levels of p65NF-κB and IL-6. The results showed that p65NF-κB and IL-6 were highly expressed in MPO-AAV rat (*P < 0.01*). Compared with the MPO-AAV group, the expresstions of p65NF-κB and IL-6 are downregulated in MPO-AAV + Anti-FKN (*P < 0.01*) (*P* < 0.05), suggesting that renal infammatory lesions were alleviated (Fig. 7).

## Discussion

In this study, we showed differential analysis and localization of chemokine FKN protein levels in a rat model of MPO-AAV-associated glomerulonephritis induced by MPO + Freund’s complete adjuvant. The results showed that the levels of FKN protein in serum and renal tissue of the rat model were significantly higher than those of the control group. Immunofluorescence analysis showed that FKN was mainly expressed in the cytoplasm of renal tubular epithelial cells and glomerular podocytes. The average optical density of FKN in MPO-AAV group was significantly higher than that in control group, while Anti-FKN could reduce the average optical density of FKN in renal tissue of MPO-AAV rats. HE staining showed that the renal tissue of MPO-AAV rats was damaged severely, however, renal function and renal tissue damage were restored to some extent by antagonizing FKN. In addition, immunohistochemical results showed that, the expression of p65NF-κB and IL-6 in MPO-AAV group were higher than that in control group, while Anti-FKN could reverse the expression of p65NF-κB and IL-6 in renal tissue of MPO-AAV rats. These results suggest that the overexpression of FKN in serum and renal tissue of MPO-AAV rats may be related to the pathogenesis of MPO-AAV associated glomerulonephritis.

Related studies have found that FKN is abnormally expressed in human kidney diseases and animal models. The expression of FKN mRNA and protein in the renal tissue of diabetic nephropathy rats was significantly higher than that of the control group, and was positively correlated with the expression level of CD68. The results showed that FKN plays a role in the inflammatory response of diabetic nephropathy [31]. Clinical studies have found that FKN is highly expressed in the blood of patients with type 2 diabetic nephropathy. Compared with healthy controls and patients with type 2 diabetes without nephropathy, the protein expression of FKN in the blood of patients with type 2 diabetes is significantly increased, so it is speculated that FKN is closely related to the occurrence and development of diabetic nephropathy [32]. The research data found that FKN was highly expressed in the renal interstitium of crescentic nephritis patients, and its expression was significantly down-regulated after glucocorticoid treatment, indicating that FKN was involved in the process of renal interstitial injury before treatment of crescentic nephritis [32]. In addition, some studies have shown that FKN is highly expressed in the blood and kidney of patients with lupus nephritis, and is related to the degree of pathological damage in renal tissue of patients with lupus nephritis [13]. Taken together, FKN plays a key role in the occurrence and development of immune-related nephropathy. Several chemokines were found to be upregulated during anti-MPO IgG-induced glomerulonephritis, and interestingly, they found that renal (mainly glomerular) cells and infiltrating inflammatory cells were in part responsible for the production of chemokines [33]. In addition, upregulation of CX3CL1 (FKN) and its receptor CX3CR1 was observed during the crescent stage [34]. As a transmembrane chemokine, CX3CL1 (FKN) is known to attract monocytes. CX3CL1 mRNA expression has been observed in glomerular lesions of patients with vascular disease [35], and in a rat model of crescentic glomerulonephritis, inhibition of CX3CR1 attenuated glomerular leukocyte influx and reduced crescent formation [36]. These data suggest that CX3CL1 (FKN) may be involved in the pathogenesis of NCGN induced by anti-MPO IgG. Consistent with their findings, FKN was highly expressed in serum and renal tissues in MPO-induced MPO-AAV-associated glomerulonephritis rats. Interestingly, the renal function and tissue damage in MPO-AAV-associated glomerulonephritis rat model were significantly restored by blocking the action of FKN.

Nuclear factor-κB (NF-κB) is a nuclear transcription factor that regulates critical cellular behavior and many cytokines by influencing biological processes of cells including inflammation, innate and adaptive immunity, and stress responses. NF-κB proteins include NF-κB2 p52/p100, NF-κB1 p50/p105, c-Rel, RelA/p65, and RelB [37]. The NF-κB pathway has been reported to be involved in the release of pro-inflammatory mediators in a variety of inflammatory diseases. For example, inhibition of NF-κB led to amelioration of acute lung injury in rats. NF-κB expression was negatively correlated with MPO levels [38]. IL-6 is a pleiotropic molecule with multiple immunologic and metabolic effects, which is a pro-inflammatory cytokine [39]. Importantly, studies have confirmed that the expression levels of FKN and IL-6 are proportional in inflammatory diseases [40]. In the present study, we found that inflammatory pathway factors p65NF-κB and IL-6 were highly expressed in MPO-AAV rat, and anti-FKN could reverse the expression of these factor, suggesting that FKN may be involved in the pathogenesis of MPO-AAV-associated glomerulonephritis through the modulation of related inflammatory signalling pathways, such as the NF-κB signalling pathway. However, the mutual regulation was not discussed in depth in this study, and the related mechanism of vitro is lacking. We will complement this research in the future to further consolidate the signifcance and value of the present study.

## Conclusions

Taken together, FKN may affect the level of kidney injury in MPO-AAV-associated glomerulonephritis rats by modulating inflammatory signalling pathways. This suggests that FKN may be a potential molecular marker for the diagnosis and treatment of MPO-AAV. However, the involvement of FKN in the pathogenesis of MPO-AAV associated glomerulonephritis and its role in the progression of the disease need to be further studied.

### Electronic supplementary material

Below is the link to the electronic supplementary material.

Supplementary Material 1

Supplementary Material 2

Supplementary Material 3

Supplementary Material 4

Supplementary Material 5

Supplementary Material 6

Supplementary Material 7

## Data Availability

The dataset supporting the conclusions of this article is included within the article.
