# Correction: expression and significance of Fractalkine/ CX3CL1 in MPO-AAV-associated glomerulonephritis rats

**DOI:** 10.1186/s12882-024-03680-1

**Published:** 2024-08-01

**Authors:** Junxue Ma, Junjie Wang, Hongli Kang, Ruiying Ma, Zhengxi Zhu

**Affiliations:** 1 Department of Nephrology, The people’s hospital of Baise, Baise, China; 2 Department of Nephrology, Cangxi People’s Hospital, Cangxi, Sichaun China; 3 Dapartment of Emergenc, The Third Hospital of Shijiazhuang, Shijiazhuang, China; 4 Department of Nephrology, The Affiliated Hospital of Youjiang Medical University for Nationalities, Baise, China

**Correction to: BMC Nephrology (2024) 25:211**

10.1186/s12882-024-03565-3

Following publication of the original article [1], the authors identified an error in the Supplementary materials.

The original article has been corrected.
