# Supplementary figures and images for: Expression and significance of Fractalkine/CX3CL1 in MPO-AAV-associated glomerulonephritis rats
Source: BMC Nephrol. 2024 Jun 27;25:211. doi: 10.1186/s12882-024-03565-3 (PMC11212253; doi:10.1186/s12882-024-03565-3)

The images of Western Blot (FKN)


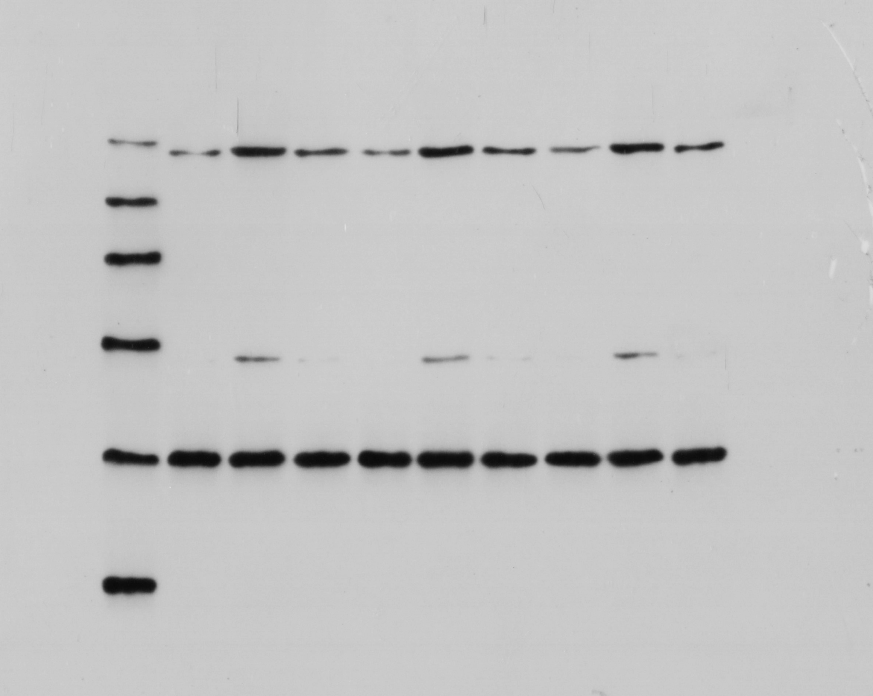


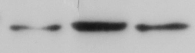

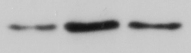

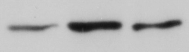

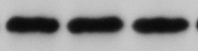

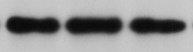

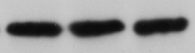


FKN-1 FKN-2 FKN-3 GAPDH-1 GAPDH-2 GAPDH-3

Supplement: Supplementary file 1 — Supplementary Material 1 [file 12882_2024_3565_MOESM1_ESM.docx]
